# Utility of Doppler-Ultrasound and Liver Elastography in the Evaluation of Patients with Suspected Pregnancy-Related Liver Disease

**DOI:** 10.3390/jcm12041653

**Published:** 2023-02-19

**Authors:** Carla Serra, Elton Dajti, Chiara De Molo, Elisa Montaguti, Alberto Porro, Anna Seidenari, Emiliana Angilletta, Vito Bernardi, Ginevra Salsi, Sofia Maria Bakken, Marco Montagnani, Giuseppe Mazzella, Francesco Azzaroli

**Affiliations:** 1 Department of Organ Insufficiency and Transplantation, IRCCS Azienda Ospedaliero-Universitaria di Bologna, 40138 Bologna, Italy; 2 Gastroenterology Unit, IRCCS Azienda Ospedaliero-Universitaria di Bologna, 40138 Bologna, Italy; 3 Department of Medical and Surgical Sciences (DIMEC), University of Bologna, 40126 Bologna, Italy; 4 Obstetric Unit, IRCCS Azienda Ospedaliero-Universitaria di Bologna, 40138 Bologna, Italy

**Keywords:** liver stiffness, elastography, Doppler-ultrasound, pre-eclampsia, liver disease, pregnancy

## Abstract

Grayscale abdomen ultrasound (US) is routinely performed in pregnant women with suspected pregnancy-related liver dysfunction, but its diagnostic yield is very low. We aimed to investigate the association between Doppler-US findings, liver stiffness measurement (LSM) and different causes of pregnancy-related liver dysfunction. This is a prospective cohort study of pregnant women referred to our tertiary center for any suspected gastrointestinal disease between 2017 and 2019 and undergoing Doppler-US and liver elastography. Patients with previous liver disease were excluded from the analysis. For group comparisons of categorical and continuous variables, the chi-square test or Mann–Whitney test, and the McNemar test were used, as appropriate. A total of 112 patients were included in the final analysis, of whom 41 (36.6%) presented with suspected liver disease: 23 intrahepatic cholestasis of pregnancy (ICP), six with gestational hypertensive disorders and 12 cases with undetermined causes of elevated liver enzymes. Values of LSM were higher and significantly associated with a diagnosis of gestational hypertensive disorder (AUROC = 0.815). No significant differences at Doppler-US or LSM were found between ICP patients and controls. Patients with undetermined causes of hypertransaminasemia showed higher hepatic and splenic resistive indexes than controls, suggesting splanchnic congestion. The evaluation of Doppler-US and liver elastography is clinically useful in patients with suspected liver dysfunction during pregnancy. Liver stiffness represents a promising non-invasive tool for the assessment of patients with gestational hypertensive disorders.

## 1. Introduction

Liver dysfunction is not uncommon during pregnancy, especially in subjects with risk factors such as twin pregnancy, obesity and previous liver disease [1,2]. The most common causes of liver dysfunction are pregnant-related liver diseases, such as hyperemesis gravidarum, intrahepatic cholestasis of pregnancy (ICP), gestational hypertension (GH), pre-eclampsia (PE) and HELLP (Hemolysis, Elevated Liver enzymes, and Low Platelet count) syndrome [1,2,3,4]. Other causes may be an exacerbation of an already present liver disease or new onset of liver disease not specific to pregnancy, such as viral hepatitis, autoimmune liver disease, metabolic dysfunction-associated liver disease etc. [1,3,5].

Abdomen ultrasound (US) is usually performed routinely in pregnant women with elevated liver enzymes and suspected liver dysfunction; however, the diagnostic yield of this technique has been questioned [6,7]. In fact, US is not usually required to establish a diagnosis of pregnancy-related liver disease, with the exception of acute fatty liver of pregnancy [1], and clinically significant US findings leading to a change in management were found only in two out of 120 (1.6%) cases in a large retrospective cohort [6]. On the other hand, other authors have suggested that maternal venous hemodynamics are dysfunctional in some pregnancy-related liver diseases, such as pre-eclampsia; therefore, its evaluation by Doppler-US may play an important role in the diagnostic work-up of these patients [8]. More recently, liver stiffness measurement (LSM), a non-invasive tool that determines the degree of liver fibrosis [9] but that also reflects hepatic congestion, necro-inflammation, and cholestasis [9], has been proposed as a useful tool in the evaluation of pregnant patients with suspected liver dysfunction [10], but these data have not yet been validated.

The present study aims to investigate the association between ultrasonoelastography findings, including Doppler-US and LSM, and different causes of pregnancy-related liver dysfunction.

## 2. Materials and Methods

### 2.1. Study Design and Population

This is a prospective cohort study conducted in our tertiary center, including all consecutive pregnant women referred to the Unit of Gastroenterology and Hepatology for any suspected gastrointestinal disease in the period between January 2017–January 2019. The participants were followed at the Obstetric Unit, IRCCS Azienda Ospedaliero-Universitaria di Bologna. All patients underwent abdominal US, including Doppler-US evaluation and LSM by two-dimensional-shear wave elastography (2D-SWE). Inclusion criteria were age ≥ 18, informed written consent, available abdominal US report and live fetus at week ≥ 20. Exclusion criteria were the presence of chronic liver disease before pregnancy and insufficient data to include in the analysis.

### 2.2. Data Collection and Definitions

For each enrolled patient, we collected data regarding previous pregnancies, gestational age, body weight, and body mass index (BMI), laboratory studies including liver enzymes and bile acid levels, Doppler-US findings, LSM values, final liver dysfunction diagnosis and fetal-perinatal outcomes, when available. The diagnosis of pregnancy-related liver diseases was established according to current recommendations [2,11,12]. Liver tests were defined as abnormal when higher than the upper limit of normal at our center (35 UI/mL). Unfavorable perinatal outcomes were defined as stillbirth, premature birth, or low birth weight.

### 2.3. Ultrasound Evaluation

All examinations were performed by a single experienced operator with GE LOGIC E9 XDclear 2.0 (GE Healthcare, Milwaukee, WI, USA) with a C1-6 convex probe. The following parameters were evaluated: flow in the three hepatic veins (HV) in supine and left lateral position, right and left hepatic artery (HA) resistive (RI) and pulsatility (PI) index, splenic artery (SA) RI and PI. We considered increased values for HA-RI and HA-PI if >0.7 and >1.2, respectively; values > 0.6 and >0.95 for SA-RI and SA-PI, respectively, were considered abnormal.

### 2.4. Liver Elastography

LSM was assessed with the ElastPQ technique, using an iU22 scanner (Philips, Bothell, WA, USA) with a convex probe C5-1. The examinations were performed in the right lobe of the liver through intercostal spaces, with the patient lying supine with the right arm in maximal abduction and suspended normal respiration. Using a real-time B-mode image, the rater selected a vessel-free area, at least 1.5 cm below the Glisson capsule, where a fixed region of interest of 0.5 × 1.5 cm was placed by moving a trackball. Using the software provided by the manufacturer, we calculated LSM expressed in kilopascal. Ten successful measurements of ElastPQ were obtained in the same location for every patient. Mean value and standard deviation within the region of interest were recorded.

### 2.5. Statistical Analysis

Categorical data are expressed as numbers (percentages), and continuous variables as medians (interquartile range, IQR). For group comparisons of categorical and continuous variables, the chi-square test or Mann–Whitney test, and the McNemar test were used, as appropriate. The association between the investigated elastosonography findings and the presence of any of the pregnancy-related liver dysfunctions among pregnant women candidates was assessed with logistic regression analyses. A 2-tailed *p*-value of <0.05 was considered statistically significant. The statistical analysis was carried out using Stata/SE (Version 14.0; Stata Corp, College Station, TX, USA).

## 3. Results

### 3.1. Patients’ Selection and Characteristics

**Figure 1:**
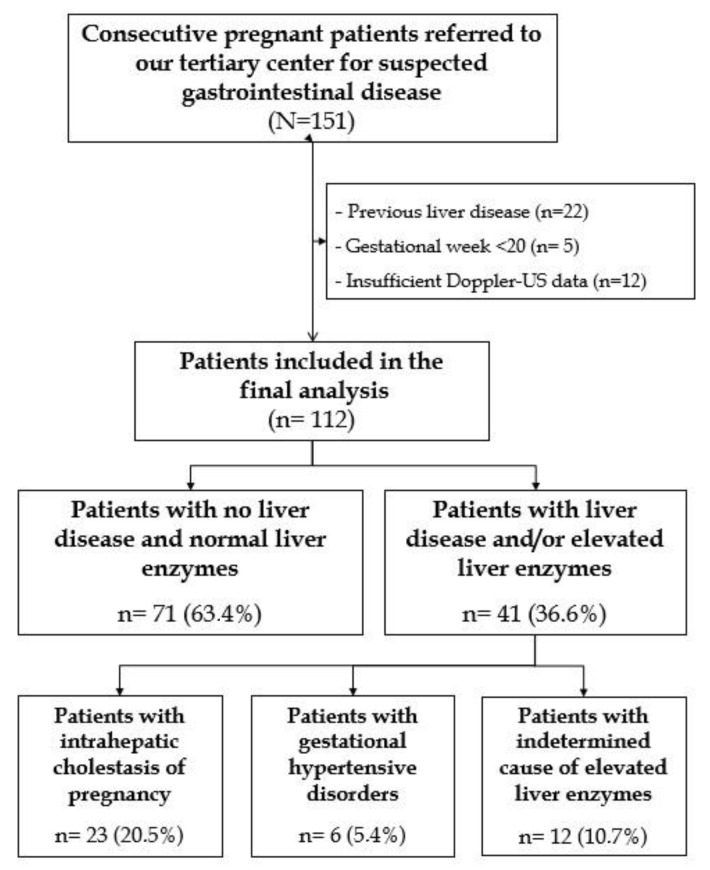
Flowchart of patients’ selection in our cohort.

A total of 151 patients were eligible for inclusion in our study. After the exclusion of 22 patients with previous chronic liver disease, five patients at gestational age < 20 weeks, and 12 for lack of Doppler-US data, a total of 112 patients were included in the final analysis (Figure 1).

The included patients had a median age of 35 (31–38) years, a median BMI of 23.9 (21–27) kg/m^2^ and were at the 34th (32nd–36th) week of gestation at enrollment (Table 1). Forty-one (36.6%) patients presented with suspected liver disease, of whom 23 were related to ICP, six to GH/PE/HELLP syndrome, and in 12 cases, the cause of elevated liver enzymes remained undetermined. Moreover, 15 (13.4%) presented gestational diabetes. Perinatal unfavorable outcomes were observed in 11 out of 100 patients with available data.

### 3.2. Role of Doppler-US and Liver Elastography in the Differential Diagnosis of Pregnancy-Related Liver Dysfunction

The study group comprised women diagnosed with ICP (*n* = 23), GH/PE/HELLP (*n* = 6), or undetermined causes of hypertransaminasemia (*n* = 12) and the control group comprised pregnant women with normal liver enzymes (*n* = 71). The clinical and elastosonography data of the two groups were compared, and the results are summarized in Table 2.

In the control group, middle HV flow was monophasic and biphasic in 24 (33.8%) and 23 (32.4%) patients, respectively; HV flow showed a similar distribution also in the right and left HV. After the decubital change in the lateral flank position (and decompression of the splanchnic circulation), the middle HV flow was triphasic in most (52, 73.2%) of the cases. Elevated indices of splanchnic flow resistance were elevated in a minority of cases, respectively, abnormal right HA-RI in 10 (14.1%) patients, left HA-RI in 16 (22.5%) patients and SA-RI in 10 (14.1%) patients. Median LSM in patients with normal liver enzymes and at a median gestational age of 35 (33–36) weeks was 4.5 (4.2–5.3) kPa.

No significant change in Doppler-US or liver elastography was found in patients with ICP compared to the control group (Table 2).

Patients with GH/PE/HELLP, on the other hand, showed statistically higher rates of patients with abnormal right HA-RI (66.7% vs. 14.1%, *p* = 0.009) and higher median LSM values (7.4 kPa vs. 4.5 kPa, *p* = 0.006).

In patients with undetermined causes of elevated liver enzymes, we found significant changes in hemodynamics and splanchnic circulation compared to controls. In particular, these patients presented significantly higher values of right HA-RI (0.60 vs. 0.70, *p* = 0.034), left HA-RI (0.63 vs. 0.71, *p* = 0.011), left HA-PI (1.02 vs. 1.44, *p* = 0.033), and SA-RI (0.54 vs. 0.60, *p* = 0.021). Of note, no difference in median LSM values was found between these two groups (*p* = 0.448).

### 3.3. Performance of Liver Elastography in the Diagnosis of Pregnancy-Related Liver Dysfunction

**Figure 2:**
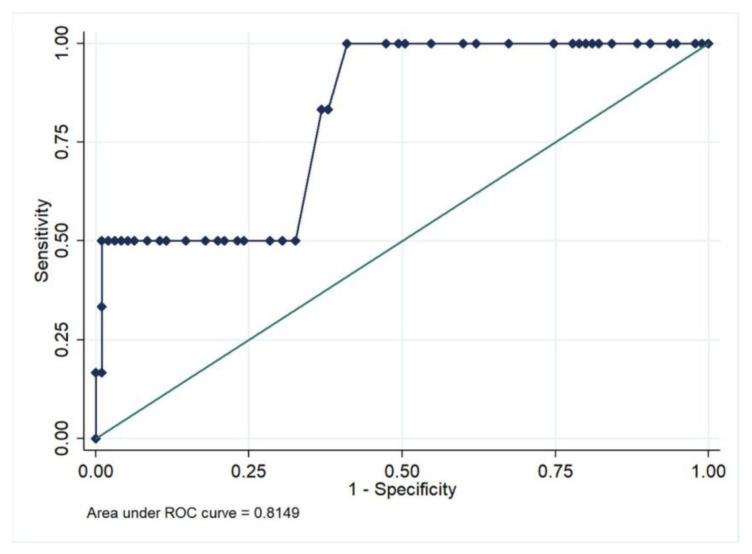
Accuracy of liver stiffness for the prediction of gestational hypertensive disorders in the overall cohort.

The LSM values were statistically associated with the diagnosis of GH/PE/HELLP, with an odds ratio of 1.875 (95%-Confidence interval; 1.191–2.950, *p*-value = 0.007) for each unit increase in LSM. The association between LSM and the diagnosis of ICP or indeterminate cause of hypertransaminasemia was not significant (*p* = 0.856 and 0.763, respectively). The AUROC of LSM values for GH/PE/HELLP diagnosis was excellent: 0.815 in the overall cohort and 0.747 in the subgroup of patients with elevated liver enzymes (Figure 2). The previously described cut-off of 7.6 kPa performed well in our cohort: the specificity and negative predictive value were high, 97.1% and 95.7%, respectively, but the sensitivity and positive predictive value were suboptimal (50% and 60%, respectively).

## 4. Discussion

In our study, we showed that Doppler-US findings and liver elastography are consistently altered in pregnant patients with suspected pregnancy-associated liver dysfunction. Higher liver stiffness values were associated with a diagnosis of gestational hypertensive disorders. Up to one-third of the patients with elevated liver enzymes did not have a conclusive diagnosis of liver disease, yet they showed significant alterations in the hemodynamics splanchnic venous system when compared to controls.

Severe hepatic dysfunction during pregnancy is rare but potentially fatal, and is associated with high morbidity and mortality, both maternal and fetal [1,3]. Therefore, a timely clinical evaluation and diagnostic work-up are crucial for the adequate management of patients with elevated liver enzymes and suspected liver disease during pregnancy. Abdomen US is almost routinely performed in these cases; however, its diagnostic yield has often been reported quite low [6,7]. We hypothesized that the inclusion of the Doppler evaluation and the measurement of liver stiffness could improve the diagnostic performance of ultrasonography and the understanding of the physiopathology of liver disease during pregnancy.

One of the main findings of our study is that values of LSM were significantly higher among patients with gestational hypertensive disorders (GP/HE/HELLP), namely 7.4 kPa vs. 4.5 kPa in controls (*p* = 0.006). The accuracy of LSM in predicting these conditions was excellent, showing an AUROC > 0.800 in the overall cohort. A previously reported cut-off of 7.6 kPa [10] was efficient in ruling-out GH/PE/HELLP (negative predictive values of 95.7%) but showed a modest positive predictive value (60%). These results are completely in line with that reported by Ammon et al. [10], the only study that has previously investigated the role of LSM in this setting. These authors also found an AUROC 0.815 for the prediction of pre-eclampsia by LSM and a similar diagnostic performance of the 7.6 kPa cut-off (sensitivity 55%, specificity 92%). Moreover, in our study, we also found that right HA-RI, a surrogate of portal flow and resistance, was increased in two-thirds of the patients with GH/PE/HELLP (vs. 14.1% in controls, *p* = 0.009).

From a pathophysiological point of view, previous studies have shown that the adaptive regulation of maternal blood volume is disturbed in pre-eclampsia, and the establishment of dysfunctional maternal venous hemodynamics with congestion plays a central role in the pathophysiology of pre-eclampsia [8]. Liver dysfunction is one of the most clinically relevant features of pre-eclampsia, and its most severe form, HELLP syndrome, is characterized by low hepatic flow and systemic inflammatory response that cause damage to hepatocytes and liver sinusoidal endothelial cells, micro-thrombi formation and fibrin deposition. All these features, hemodynamic (congestion) and non (liver inflammation, necrosis and endothelial damage), could determine an increase in the pressure exerted upon the liver capsule and could be accurately reflected by the evaluation of LSM, as shown for many other similar conditions that share partly the same underlying pathogenetic mechanisms [13,14,15,16]. From a clinical point of view, LSM represents a promising simple and non-invasive tool that could quickly be offered to all patients with elevated liver enzymes or suspected gestational hypertensive disorder during pregnancy to evaluate the presence and eventually the severity of these disorders. So, future studies are warranted to confirm and further explore the diagnostic and prognostic significance that this test can bare in this context.

Regarding ICP, we found no difference in median LSM values among patients with ICP and controls (5 kPa vs. 4.5 kPa, *p* = 0.195); the rate of patients with high LSM values (≥7.6 kPa) was numerically but not statistically significant, higher in the ICP group (11.8% vs. 2.9%, *p* = 0.120), This is slightly in contrast with the study by Amonn et al., where LSM values in ICP patients were significantly higher than in controls (6.8 kPa vs. 5.3 kPa). From a theoretical point of view, intrahepatic cholestasis is another condition that can determine an increase in liver stiffness [9,17], so slightly higher LSM values are plausible in ICP patients. Noteworthy, we found no significant differences in any of the evaluated Doppler-US parameters between patients with ICP and controls, suggesting that venous hemodynamics do not play a significant role in the development of ICP and that the evaluation with Doppler-US could help in the differential diagnosis between cholestatic and hypertension-related disorders during pregnancy.

Finally, the cause for elevated liver enzymes can remain undetermined in a significant proportion of patients (29.3% in our cohort). The most novel finding of this study is that Doppler-US parameters evaluating splanchnic hemodynamics showed profound differences between these patients and controls, as multiple parameters evaluating both hepatic (right HA-RI, left-HA-RI, left HA-PI) and splenic (SA-RI) vascular resistance were higher in patients with unknown cause for elevated liver enzymes. One explanation could be that liver enzymes in these patients reflect the above-mentioned maladaptation of the splanchnic circulation to the increased circulating volumes occurring during pregnancy, but these changes remain subclinical or pre-pathological and do not result in the development of clinically overt hypertensive disorders as in PE/HELLP. Another cause for transiently elevated liver enzymes could be metabolic dysfunction, determined by pre-existent obesity or gestational diabetes, but in this case, one would not expect the profound changes in Doppler-US parameters found in these patients.

Our study has some limitations. Firstly, the tertiary nature of our hepatological and obstetrics center determined a relatively high prevalence of patients with liver dysfunction during pregnancy included in the study, and this may limit the generalizability of our results outside this context. Moreover, the low number of patients with gestational hypertensive disorders did not allow us to fully evaluate the differences in LSM and Doppler-US parameters separately for GS, early- and late-onset PE, and HELLP syndrome. However, another large prospective study is currently ongoing in our center to further address these issues.

In conclusion, the evaluation of Doppler-US and liver elastography is clinically useful in patients with suspected liver dysfunction during pregnancy. Liver stiffness measurement represents a promising non-invasive tool for the assessment of patients with gestational hypertensive disorders. Hepatic congestion, as suggested by Doppler-US findings, might explain the rise in liver enzymes in patients who do not have a conclusive diagnosis of liver disease during pregnancy. Further large prospective studies are warranted to validate our results and further explore the potential role of liver elastosonography in this clinical context.

## Figures and Tables

**Table 1:** Characteristics of the patients included in the study.

Variables	All Patients (*n* = 112)	Patients with No Liver Disease (*n* = 71)	Patients with Liver Disease (*n* = 41)	*p*-Value
Age (years)	35 (31–38)	34 (30–38)	36 (32–38)	0.370
BMI (kg/m^2^)	23.9 (21.4–27)	23 (21.3–25.4)	25 (21.5–28)	0.113
Overweight	52 (46.4%)	28 (39.4%)	24 (58.5%)	*0.051*
Relative weight gain (%)	18.6 (14.4–25.4)	20.6 (16.4–28.1)	15 (11.3–22)	**0.039**
Week of gestation	34 (32–36)	35 (33–36)	33 (29–36)	**0.040**
Parity	1 (1–1)	1 (1–2)	1 (1–1)	**0.038**
**Biochemical tests**				
AST (UI/L)	21 (17–38)	19 (15–23)	46 (39–75)	**<0.0001**
ALT (UI/L)	18 (11–45)	14 (10–21)	79 (41–126)	**<0.0001**
Bilirubin (mg/dL)	0.42 (0.36–0.55)	0.41 (0.36–0.52)	0.43 (0.35–0.58)	0.651
γ-GT (UI/L)	12 (8–19)	12 (8–18)	14 (9–20)	0.125
Bile acid levels (µmol/L) (*n* = 78)	6.7 (3–15.5)	3.2 (2.1–6)	16 (9.3–24.1)	**<0.0001**
**Liver-related dysfunction**				
Elevated liver enzymes	41 (36.6%)			
Intrahepatic cholestasis of pregnancy	23 (20.5%)			
Hypertension/pre-eclampsia/ HELLP syndrome	6 (5.4%)			
Unknown	12 (10.7%)			
**Metabolic complications**				
Gestational diabetes	15 (13.4%)	7 (9.9%)	8 (19.5%)	0.148
**Perinatal outcomes**				
Birth weight (g)	3050 (2740–3380)	3050 (2800–3380)	3085 (2695–3383)	0.547
Unfavourable outcomes (*n* = 100)	11 (11%)	5 (7.8%)	6 (16.7%)	0.174

ALT: alanine transaminase; AST: aspartate transaminase BMI: body mass index; γ-GT: gamma-glutamyl transferase; HELLP: Hemolysis, Elevated Liver enzymes, and Low Platelet count.

**Table 2:** Characteristics of the pregnant patients with normal liver enzymes and with pregnancy-related liver dysfunction.

Variables	Patients with Normal Liver Enzymes (n = 71)	Patients with ICP (n = 23)	*p*-Value	Patients with GH/PE/HELLP (n = 6)	*p*-Value	Patients with Undetermined Causes of Elevated Liver Enzymes (n = 12)	*p*-Value
Age (years)	34 (30–38)	36 (32–38)	0.303	35 (34–35)	0.900	35 (31–39)	0.747
BMI (kg/m^2^)	23 (21.3–25.4)	25.7 (22.1–29.2)	0.136	21.4 (21.1–24.5)	0.404	26.8 (24.8–26.9)	*0.053*
Overweight	28 (39.4%)	14 (60.9%)	*0.072*	1 (16.7%)	0.269	9 (75%)	**0.024**
Relative weight gain (%)	20.6 (16.4–28.1)	11.9 (9.8–18.7)	**0.005**	19.7 (15.9–29.7)	0.917	20.3 (16.4–29.4)	0.917
Week of gestation	35 (33–36)	33 (29–36)	0.156	33 (33–33)	0.188	33 (27–35)	0.150
Parity	1 (1–2)	1 (1–1)	**0.035**	1 (1–2)	0.443	1 (0–1)	0.115
Gestational diabetes	7 (9.9%)	4 (17.4%)	0.328	1 (16.7%)	0.600	3 (25%)	0.136
**Perinatal outcomes**							
Unfavorable outcomes	5 (7.8%)	3 (14.3%)	0.378	2 (33.3%)	0.107	1 (11.1%)	0.736
**Doppler-US findings**							
Middle-HV flow			0.831		0.998		0.225
Monophasic	24 (33.8%)	9 (39.1%)		2 (33%)		6 (50%)	
Biphasic	23 (32.4%)	6 (26.1%)		2 (33%)		1 (8.3%)	
Triphasic	24 (33.8%)	8 (34.8%)		2 (33%)		5 (41.7%)	
Middle-HV flow in the lateral position			0.702		0.449		0.126
Monophasic	8 (11.3%)	4 (17.4%)		1 (16.7%)		4 (33.3%)	
Biphasic	11 (15.5%)	4 (17.4%)		2 (33.3%)		1 (8.3%)	
Triphasic	52 (73.2%)	15 (65.2%)		3 (50%)		7 (58.4%)	
Improvement in HV flow after lateral position	35 (49.3%)	10 (43.5%)	0.627	2 (33%)	0.676	3 (25%)	0.209
Right HA-RI	0.60 (0.57–0.67)	0.61 (0.59–0.69)	0.342	0.73 (0.64–0.87)	*0.070*	0.70 (0.66–0. 75)	**0.034**
Right HA-RI > 0.7 (yes)	10 (14.1%)	6 (26.1%)	0.208	4 (66.7%)	**0.009**	8 (66.7%)	**0.003**
Left HA-RI	0.63 (0.58–0.69)	0.60 (0.58–0.71)	0.880	0.63 (0.57–0.69)	0.990	0.71 (0.70–0.74)	**0.011**
Left HA-RI > 0.7 (yes)	16 (22.5%)	9 (39.1%)	0.173	2 (33.3%)	0.620	10 (83.3%)	**0.001**
Right HA-PI	0.97 (0.85–1.17)	1.10 (0.93–1.33)	0.104	1.12 (0.90–1.52)	0.355	1.19 (1.10–1.44)	0.165
Right HA-PI > 1.2 (yes)	54 (76.1%)	20 (87%)	0.383	6 (100%)	0.329	11 (91.7%)	0.448
Left HA-PI	1.02 (0.86–1.30)	0.96 (0.91–1.49)	0.513	1.04 (0.97–1.38)	0.596	1.44 (1.30–1.45)	**0.033**
Left HA-PI > 1.2 (yes)	24 (33.8%)	10 (47.6%)	0.458	2 (33.3%)	0.981	8 (66.7%)	*0.051*
SA-RI	0.54 (0.51–0.58)	0.54 (0.51–0.59)	0.826	0.54 (0.51–0.58)	1	0.60 (0.57–0.72)	**0.021**
SA-RI > 0.6 (yes)	10 (14.1%)	3 (14.3%)	0.390	1 (16.7%)	0.862	5 (41.7%)	**0.037**
SA-PI	0.80 (0.72–0.87)	0.79 (0.72–0.92)	0.962	0.76 (0.71–0.88)	0.794	0.90 (0.70–0.95)	0.594
SA-PI > 0.95 (yes)	9 (12.7%)	2 (8.7%)	0.886	1 (16.7%)	0.579	2 (16.7%)	0.657
**Elastography (*n* = 101)**							
Liver stiffness (kPa)	4.5 (4.2–5.3)	5 (4.3–5.5)	0.195	7.4 (5.1–9.9)	**0.006**	5.1 (4.2–5.7)	0.448
Liver stiffness ≥ 7.6 kPa	2 (2.9%)	2 (11.8%)	0.120	3 (60%)	**0.003**	0 (0%)	0.605

BMI: body mass index; GH: gestational hypertension; HA: hepatic artery; HELLP: Hemolysis, Elevated Liver enzymes, and Low Platelet count; HV-hepatic vein; ICP: intrahepatic cholestasis of pregnancy; PH: pregnancy-related hypertension; PI: pulsatility index; RI: resistive index; SA: splenic artery.

## Data Availability

Data are available on request from the authors.
